# Three-year impact of COVID-19 pandemic on hospitalized twin pregnancies: evaluation of characteristics and changes in antibiotic prescribing

**DOI:** 10.3389/fmed.2025.1546013

**Published:** 2025-06-18

**Authors:** Qin-Yu Cai, Shang Jing Liu, Xing-Qi Zhi, Wei-Zhen Tang, Ying-Xiong Wang, Xia Lan, Li Wen, Shu-Juan Luo, Lan Wang, Jie Sheng, Tai-Hang Liu

**Affiliations:** ^1^Department of Bioinformatics, School of Basic Medical Sciences, Chongqing Medical University, Chongqing, China; ^2^Department of Obstetrics, Women and Children’s Hospital of Chongqing Medical University, Chongqing, China; ^3^Joint International Research Laboratory of Reproduction and Development, Chongqing Medical University, Chongqing, China; ^4^Department of Pathology, China-Japan Friendship Hospital, Beijing, China

**Keywords:** COVID-19 pandemic, retrospective cohort study, twin pregnancies, clinical characteristics, antibiotic prescribing

## Abstract

**Background:**

The COVID-19 pandemic has significantly impacted healthcare systems worldwide, including obstetric care. However, the long-term effects on twin pregnancies remain unclear. This study investigates the impact of COVID-19 on the clinical characteristics and antibiotic prescribing patterns in hospitalized twin pregnancies.

**Methods:**

A retrospective cohort study was conducted at Women and Children’s Hospital of Chongqing Medical University, Chongqing, China, involving 3,827 twin pregnancies with live deliveries between 1 January 2017 and 31 December 2022. The pre-pandemic group included 1,707 patients, and the pandemic group included 2,120. Sociodemographic and clinical data were analyzed using general linear models with SPSS and R software.

**Results:**

During the pandemic, twin pregnancy admissions increased by 24.19%. Patients in the pandemic group have less gestational weight gain (17.00 vs. 16.08 kg, *P* < 0.001), had higher rates of assisted reproductive technology use (73.2% vs. 68.7%, *P* = 0.002), and experienced more complications. Neonates showed higher rates of pneumonia (5.7% vs. 3.8%, *P* < 0.001) and NICU admissions (43.7% vs. 13.9%, *P* < 0.001). Longer hospital stays were observed in the pandemic group (*P* = 0.004). Antibiotic prescriptions, especially non-repeat prescriptions, increased for older patients, those with higher BMI, and premature deliveries. The rate of repeated antibiotic prescriptions for bacterial vaginosis increased 1.68 times.

**Conclusion:**

COVID-19 influenced twin pregnancy admissions, clinical characteristics, and antibiotic use. The study highlights the need for rational antibiotic use and improved healthcare resource management in future crises.

## Introduction

Coronavirus Disease 2019 (COVID-19), caused by the severe acute respiratory syndrome coronavirus 2 (SARS-CoV-2), was declared a pandemic on 11 March 2020 by the World Health Organization (1, 2). In many countries, hospital capacity and existing healthcare resources have been overwhelmed by the influx of patients with SARS-CoV-2 infection. The COVID-19 pandemic has had a profound impact on the global healthcare system, disrupting a variety of health care services for non-COVID-19 diseases and conditions, with particular implications for vulnerable populations, including the perinatal population (3, 4). A preliminary review showed a significant increase in obstetric personnel absences during the COVID-19 pandemic in New York City (5). Previous studies revealed that the pandemic caused a decline in maternal and neonatal health services in many countries, such as Italy (6), Nepal (7) and the United Kingdom (8). As the first country to be affected by COVID-19, obstetrics in China have also been seriously impacted. A study covering a representative sample of 11,806 physicians actively practicing obstetrics and gynecology in 779 hospitals from 157 cities across 31 provinces in China showed that during the COVID-19 pandemic, clinical obstetrics and gynecology activities in China markedly decreased, with significant differences across municipalities and hospital characteristics (9).

Due to physiological, mechanical, and immunological changes during pregnancy, pregnant women are more likely to experience severe disease. Current evidence suggests that pregnant women may face an increased risk for more severe COVID-19 disease (10), and those with moderate to severe COVID-19 are associated with adverse perinatal outcomes (4, 11). Although twin pregnancies account for a small proportion of all pregnancies, they carry a significantly higher risk than singleton pregnancies, with stillbirth rates increased 13-fold in monochorionic twins and fivefold in dichorionic twins (12, 13). Besides, twin pregnancies are more likely to experience obstetric complications such as preterm birth, intrauterine growth restriction, preeclampsia, and higher rates of cesarean delivery (14). Twin pregnancies have become more prevalent in recent years due to the increasing use of assisted reproductive technologies (ART) (15), further highlighting their public health relevance.

Antimicrobial resistance (AMR) is a growing threat to global health. The impact of COVID-19 on global AMR has been much debated in recent literature and likely varies greatly across diseases and populations (16). On the one hand, the reduction in person-to-person contact and changes in healthcare practices during the pandemic—such as the widespread use of personal protective equipment, travel restrictions, avoidance of hospitals and clinics, online consultations, and environmental disinfection—may alleviate some of the impact of COVID-19-related antibiotic use on AMR (17). On the other hand, despite relatively low rates of bacterial or fungal co-infections, antibiotics were often prescribed disproportionately in COVID-19 patients, potentially exacerbating the AMR problem (18–20). Repeat prescribing involves long-term repeat prescriptions for chronic conditions as well as the renewal of short-term antibiotic prescriptions for acute issues that persist beyond a single course of treatment (21). Previous studies have shown that repeat prescribing is unlikely to confer clinical benefit for acute conditions such as respiratory tract infections (22). Therefore, reducing inappropriate antibiotic use remains a global priority. Although prior studies have reported a substantial decline in antibiotic prescribing during the pandemic in both pediatric (23) and adult primary care settings (24), such trends may not extend to all populations. Twin pregnancies, for example, present unique challenges. Immunological adaptations in these pregnancies increase vulnerability to infections, while higher rates of obstetric complications, frequent hospitalizations, and surgical interventions necessitate more intensive and often cautious clinical management. These factors collectively contribute to a greater reliance on antibiotic use in this high-risk group, underscoring the need to better understand their antibiotic prescribing patterns.

Most studies to date have either excluded twin pregnancies or analyzed them in combination with singleton pregnancies, thereby overlooking the unique risks and healthcare needs of this population (25). By focusing specifically on hospitalized twin pregnancies during the COVID-19 pandemic, this study seeks to fill a critical gap in the literature. In this study, we explore the 3 years impact of the COVID-19 pandemic on twin pregnancy hospitalizations, maternal complications, neonatal outcomes, and antibiotic prescribing patterns. Our findings will facilitate better preparation for future public health emergencies and inform future antibiotic stewardship interventions to improve prescribing practices.

## Materials and methods

### Study design and population

**FIGURE 1:**
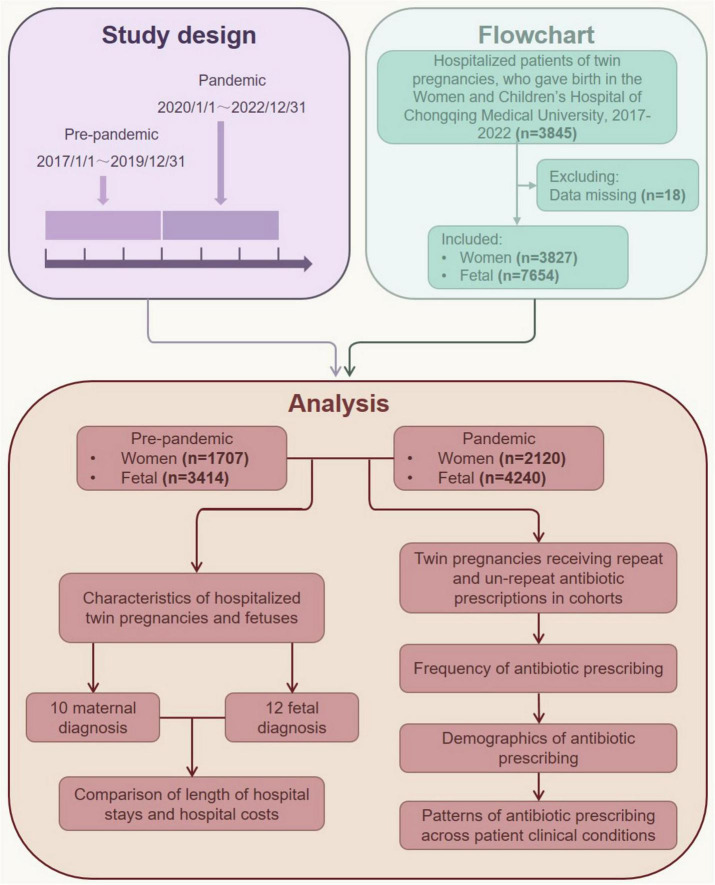
Overview of the study.

This retrospective cohort study was conducted at the Women and Children’s Hospital of Chongqing Medical University, the largest maternity hospital in Chongqing, southwest China, with an annual total of over 15,000 newborns. The study period spanned from 1 January 2017, to 31 December 2022 (Figure 1). We included all hospitalized twin pregnancies in our study if they met the following criteria: (1) the patients were hospitalized with twin pregnancies and gave birth at the Women and Children’s Hospital of Chongqing Medical University, and (2) their hospital stay was longer than 1 day. (3) Patients without known COVID-19 infections or those admitted for postpartum reasons. Patients who did not meet these criteria or had missing data over 5% were excluded from the study. Given that public health policies for the COVID-19 pandemic in China started in January 2020 and ended in December 2022, patients hospitalized from 1 January 2020 to 31 December 2022, were grouped as the pandemic cohort, while those hospitalized from 1 January 2017 to 31 December 2019, were grouped as the pre-pandemic cohort (Figure 1). The definition of each wave of the COVID-19 pandemic was based on data from the Chongqing Health Commission^1^.

Patient clinical conditions related to antibiotic use were categorized based on indications (infections treated with antibiotics). The following most common indications in twin pregnancies were investigated: upper respiratory tract infection, urinary tract infection, and bacterial vaginosis. Antibiotic prescribing in cohorts was measured using a 6 months lookback period. The number of antibiotics prescribed during the 6 months period before the cohort index date was calculated to create a primary molecule variable, from which binary categorical variables for repeat and single prescriptions were derived. Specifically, repeat antibiotic prescribing was defined as patients receiving three or more prescriptions for a specific antibiotic class during the 6 months review period. Non-repeat prescribing was defined as 1–2 antibiotic prescriptions (same antibiotic class) within the 6 months lookback period. In addition to assessing the number of antibiotics prescribed in the 6 months lookback period, the date of the first prescription within this window was also recorded and subsequently used to derive the clinical condition indication variables. If a patient’s first prescription date was missing, this information was marked as missing. Additionally, we used a patient cohort from 1 January 2017 to 31 December 2022, to determine monthly rates of repeat and non-repeat antibiotic prescriptions.

### Sociodemographic and clinical variables

The sociodemographic and clinical characteristics of patients were obtained from the Electronic Medical Record Systems of the Women and Children’s Hospital of Chongqing Medical University. These characteristics included age, gestational age, gestational weight gain, pre-pregnancy body mass index, and other baseline data, as well as the Tenth Revision (ICD-10) code of the primary discharge diagnosis for both mother and fetus, length of hospital stay, and total hospital cost. All outcome measures adhere to extensive previous research on comparative programs and national guidelines. For example: HDP is defined by systolic blood pressure of ≥ 140 mm Hg and/or diastolic blood pressure of ≥ 90 mm Hg, measured on at least two separate occasions, occurring after 20 weeks of gestation, and without significant proteinuria, as defined by the International Association for the Study of Hypertension in Pregnancy (ISSHP) (29899139). Preeclampsia, on the other hand, was diagnosed when HDP was accompanied by new-onset proteinuria or end-organ dysfunction. GDM was diagnosed when a pregnant woman underwent the 75 g OGTT during 24th–28th weeks of pregnancy and her blood glucose value met any one of the three criteria: “≥ 5.1 mmol/L (fasting), ≥ 10.0 mmol/L (1 h), or ≥ 8.5 mmol/L (2 h),” excluding having pre-pregnancy diabetes. In the cross-sectional study, GDM was defined as pregnant women having “fasting blood glucose ≥ 5.1 mmol/L.” The diagnostic criteria of neonatal pneumonia: most patients had a fever, air irritation, and cough, and fixed wet rales could be heard on pulmonary audiometry or pneumonia changes confirmed by pulmonary imaging. Based on a previous study (26), hospital costs were adjusted using the 2017–2023 Chongqing Consumer Price Index for medical care, as reported by the Chongqing Bureau of Statistics (Supporting Information: Supplementary Table 1).

### Statistical analysis

Continuous variables with a normal distribution, including maternal age, gestational age, and gestational weight gain, were presented as mean ± SD. Categorical variables, such as body mass index, fetal diagnosis, and other sociodemographic and obstetric histories, were described using frequencies and percentages. A general linear model was used to compare the frequency of different patient groups with the pre-pandemic group. The main outcome indicators in our study are quantitative data, so before the analysis, we conducted visual checks (such as Q-Q plots) and statistical tests (such as Shapiro-Wilk tests) on all continuous variables to ensure that they conformed to a normal distribution. For variables that do not conform to the normal distribution, we adopted logarithmic transformation or other appropriate transformation methods to improve the data distribution before regression analysis. The covariates included in the regression models were maternal age, gestational age, gestational weight gain, pre-pregnancy body mass index, nulliparity, ART, and 10 disease groups (for “Total” only). Unless otherwise stated, *P*-values are unadjusted for multiple testing. When analyzing specific disease categories, Bonferroni’s α = 0.05 (two-tailed) was used to adjust the significance level by dividing 0.05 by the number of disease categories (i.e., α = 0.05/10 = 0.005). The rate of repeat and non-repeat antibiotic prescribing was calculated per total study population. Monthly changes in prescription rates during the COVID-19 pandemic were calculated by subtracting pre-pandemic levels to exclude the influence of season. We also assessed changes in repeat and non-repeat prescription rates during the pandemic compared to the pre-pandemic cohort, stratified by demographic characteristics. Antibiotic prescribing patterns were analyzed for the three most common infections in twin pregnancies. The relative frequency and percent change of antibiotic prescribing rate of these three clinical infections were examined. Missing data in the statistical analysis variables did not exceed 5%, and multiple imputation was employed to account for missing values, incorporating baseline efficacy variables. The main outcome indicators in our study are quantitative data, so before the analysis, we conducted visual checks (such as Q-Q plots) and statistical tests (such as Shapiro-Wilk tests) on all continuous variables to ensure that they conformed to a normal distribution. For variables that do not conform to the normal distribution, we adopted logarithmic transformation or other appropriate transformation methods to improve the data distribution.

All data were analyzed using the Statistical Package for the Social Sciences 27.0 software (SPSS 27.0, IBM Corporation, Chicago, Illinois, United States) and R software (Version 4.4.1, Vienna, Austria). Significance was defined as a two-sided *P*-value < 0.05.

## Results

### Monthly twin pregnancy hospitalizations

**FIGURE 2:**
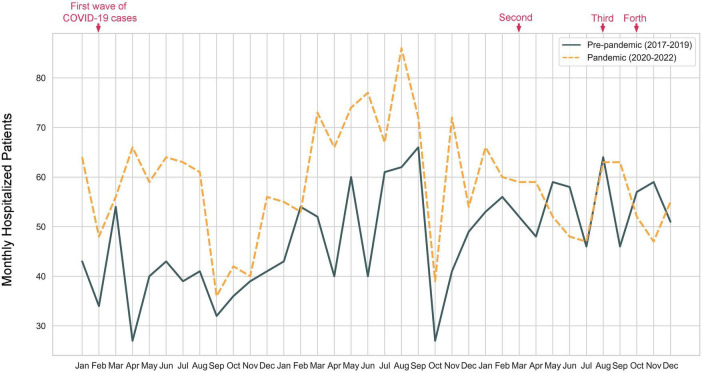
Number of monthly hospitalizations for twin pregnancies from 1 January 2017 to 31 December 2022.

A total of 3,827 twin pregnancy inpatients who met the criteria were included in the study, comprising 1,707 patients (44.6%) in the pre-pandemic cohort (hospitalized between 1 January 2017 and 31 December 2019) and 2,120 patients (55.4%) in the pandemic cohort (hospitalized between 1 January 2020 and 31 December 2022) (Figure 1). The monthly counts of twin pregnancy inpatients were further analyzed (Figure 2). Overall, the number of twin pregnancy patients admitted to the hospital was higher in the pandemic group and gradually returned to pre-pandemic levels starting in April 2022. The total number of hospitalized patients in the pandemic group increased by 24.2%, compared with the pre-pandemic group (Table 1). Specifically, during the first 2 years of the COVID-19 pandemic, we observed a marked increase in the number of twin pregnancy hospitalizations, with admissions peaking in August 2021. We found that the monthly count of twin pregnancy hospitalizations seems to be unrelated to the waves of the COVID-19 pandemic.

**TABLE 1:** Characteristics of hospitalized twin pregnancies and fetuses.

Characteristics	Total (2017–2022)	Pre-pandemic (2017–2019)	Pandemic (2020–2022)	*P*-value
**Maternal**				
No. of cases	3,827	1,707	2,120	–
Ages, years	30.91 ± 3.90	30.87 ± 3.88	31.85 ± 3.87	0.210
Gestational age	35.52 ± 2.75	35.71 ± 2.19	35.37 ± 3.12	< 0.001***
**Age groups (years)**				
< 35	3,186 (83.3%)	1,415 (82.9%)	1,771 (83.5%)	–
≥ 35	641 (16.7%)	292 (17.1%)	349 (16.5%)	–
Gestational weight gain, kg	16.49 ± 5.60	17.00 ± 5.23	16.08 ± 5.85	< 0.001***
**Body mass index before pregnancy**				0.053
< 18.5	443 (11.6%)	217 (12.7%)	226 (10.7%)	–
18.5–23.9	2,622 (68.5%)	1,132 (66.3%)	1,490 (70.3%)	–
24–27.9	654 (17.1%)	310 (18.2%)	344 (16.2%)	–
≥ 28	108 (2.8%)	48 (2.8%)	60 (2.8%)	–
**Nulliparity**				0.598
Yes	3,137 (82.0%)	1,393 (81.6%)	1,744 (82.3%)	–
No	690 (18.0%)	314 (18.4%)	376 (17.7%)	–
**Assisted reproductive technology**				0.002**
Using	2,724 (71.2%)	1,172 (68.7%)	1,552 (73.2%)	–
No using	1,103 (28.8%)	535 (31.3%)	568 (26.8%)	–
**Chorionicity**				0.618
Dichorionic	3,115 (81.4%)	1,391 (81.5%)	1,724 (81.3%)	–
Monochorionic	712 (18.6%)	316 (18.5%)	396 (18.7%)	–
**Diagnosis**				
Hypertension disorders of pregnancy	213 (5.6%)	83 (4.9%)	130 (6.1%)	0.089
Pre-eclampsia	500 (13.1%)	204 (12.0%)	296 (14.0%)	0.066
Gestational diabetes mellitus	1,070 (28.0%)	470 (27.5%)	600 (28.3%)	0.599
Intrahepatic cholestasis of pregnancy	597 (15.6%)	252 (14.8%)	345 (16.3%)	0.200
Gestational hyperthyroidism	27 (0.7%)	14 (0.8%)	13 (0.6%)	0.447
Gestational hypothyroidism	365 (9.5%)	144 (8.4%)	221 (10.4%)	0.021*
Gestational anemia	997 (26.1%)	447 (26.2%)	550 (25.9%)	0.865
Thalassemia α	94 (2.5%)	42 (2.5%)	52 (2.5%)	0.952
Thalassemia β	59 (1.5%)	27 (1.6%)	32 (1.5%)	0.897
Gestational Thrombocytopenia	185 (4.8%)	58 (3.4%)	127 (6.0%)	< 0.001***
**Fetal**				
No. of cases	7,654	3,414	4,240	–
**Diagnosis**				
1 min Apagar < 7	129 (1.7%)	70 (2.1%)	59 (1.4%)	0.352
Admission to pediatrics	4,985 (65.2%)	2,557 (74.9%)	2,428 (57.3%)	< 0.001***
Admission to NICU	2,326 (30.4%)	473 (13.9%)	1,853 (43.7%)	< 0.001***
Hypoglycemia	228 (3.0%)	113 (3.3%)	115 (2.7%)	0.128
Hyperbilirubinemia	1,054 (13.8%)	436 (12.8%)	618 (14.6%)	0.733
Respiratory distress	507 (6.6%)	228 (6.7%)	279 (6.6%)	0.840
Respiratory failure	444 (5.8%)	216 (6.3%)	228 (5.4%)	0.144
Pneumonia	369 (4.8%)	128 (3.8%)	241 (5.7%)	< 0.001***
Lower gastrointestinal bleeding	186 (2.4%)	73 (2.1%)	113 (2.7%)	0.831
Hypoproteinemia	110 (1.4%)	55 (1.6%)	55 (1.3%)	0.440
Hyperelastic acidemia	169 (2.2%)	82 (2.4%)	87 (2.1%)	0.517
Necrotizing enterocolitis	82 (1.1%)	39 (1.1%)	45 (1.1%)	0.956
Length of hospital stay, days	4 (3, 5)	4 (4, 5)	4 (3, 5)	< 0.001***
Hospital costs, RMB^a^	11446.02 ± 4078.71	11438.74 ± 3327.39	11452.69 ± 4663.65	0.577

*^a^*The results were corrected by the consumer price index (CPI) in Chongqing, China (see Supporting Information: Supplementary Table 1 for details). *P < 0.05; **P < 0.01; ***P < 0.001. Data are *n* (%) or mean (SD) or median (25th–75th percentile) unless otherwise specified.

### Characteristics of twin pregnancy inpatients and their fetuses

Our result showed that the hospitalized patients in the pandemic group were generally have less gestational weight gain (17.00 vs. 16.08 kg, *P* < 0.001, pandemic cohort vs. pre-pandemic cohort) (Table 1). We observed a higher proportion of complications in the pandemic group, including hypertension disorders of pregnancy (6.1% vs. 4.9%), pre-eclampsia (14.0% vs. 12.0%), gestational diabetes mellitus (28.3% vs. 27.5%), intrahepatic cholestasis of pregnancy (16.3% vs. 14.8%), while gestational hypothyroidism (10.4% vs. 8.4%, *P* = 0.021) and gestational Thrombocytopenia (6.0% vs. 3.4%, *P* < 0.001) have great significant differences. Additionally, the pandemic group had a higher proportion of ART use (73.2% vs. 68.7%, *P* = 0.002). For neonates, there was an increase in the rates of pneumonia (5.7% vs. 3.8%, *P* < 0.001) and NICU admissions (43.7% vs. 13.9%, *P* < 0.001).

The impact of the COVID-19 pandemic on the length of hospital stays and hospital costs was further analyzed (Table 2). The median length of hospital stay was 4 days in both groups. However, general linear models indicated that patients in the pandemic group had longer hospital stays compared to those in the pre-pandemic group (β = 0.581, SE = 0.204, *P* = 0.004), even after adjusting for age, gestational age, gestational weight gain, pre-pregnancy BMI, nulliparity, ART, and 10 disease groups (β = 0.857, SE = 0.214, *P* < 0.001). Specifically, patients with gestational thrombocytopenia had longer hospital stays in the pandemic group (β = 0.556, SE = 0.163, *P* < 0.001), even after adjustment (β = 0.647, SE = 0.163, *P* < 0.001). Regarding hospital costs, the mean costs were similar for both groups (¥11,452.69 vs. ¥11,438.74). General linear models confirmed this conclusion (β = 0.259, SE = 0.266, *P* = 0.330), even after adjustment (β = 0.280, SE = 0.268, *P* = 0.297). However, the pandemic group had a higher standard deviation, indicating greater individual differences in hospitalization costs (¥11,452.69 ± 4,663.65 vs. ¥11,438.74 ± 3,327.39). Moreover, for fetal lengths of hospital stays and hospital costs, general linear models showed no statistical differences between patients in the pandemic group and those in the pre-pandemic group (*P* = 0.067 for hospital stay length, *P* = 0.307 for hospital costs, Supplementary Table 2).

**TABLE 2:** Comparison of length of hospital stays and hospital costs for maternal diagnosis.

	Length of hospital stays			Hospital costs		
	β	SE	*P^a^*	β	SE	*P* ^a^
**Model 1**						
Total	0.581	0.204	**0.004**	−0.259	0.266	0.330
Hypertension disorders of pregnancy	0.256	0.146	0.079	0.221	0.151	0.143
Pre-eclampsia	0.198	0.100	0.047	0.153	0.102	0.134
Gestational diabetes mellitus	0.020	0.073	0.784	0.020	0.076	0.795
Intrahepatic cholestasis of pregnancy	0.119	0.092	0.195	0.105	0.095	0.269
Gestational hyperthyroidism	−0.338	0.389	0.385	−0.342	0.422	0.417
Gestational hypothyroidism	0.223	0.113	0.049	0.215	0.117	0.065
Gestational anemia	−0.002	0.075	0.645	−0.009	0.078	0.303
Thalassemia α	0.412	0.893	0.583	1.174	1.140	0.293
Thalassemia β	0.497	0.906	0.979	1.217	1.153	0.909
Gestational Thrombocytopenia	0.556	0.163	**< 0.001**	0.574	0.167	**< 0.001**
**Model 2**						
Total	0.857	0.214	**< 0.001**	−0.280	0.268	0.297
Hypertension disorders of pregnancy	0.281	0.146	0.055	0.245	0.151	0.105
Pre-eclampsia	0.333	0.102	**0.001**	0.268	0.104	0.010
Gestational diabetes mellitus	0.007	0.075	0.928	0.007	0.077	0.925
Intrahepatic cholestasis of pregnancy	0.117	0.092	0.205	0.097	0.095	0.307
Gestational hyperthyroidism	−0.258	0.389	0.507	−0.247	0.421	0.556
Gestational hypothyroidism	−0.237	0.113	0.037	0.230	0.117	0.049
Gestational anemia	−0.046	0.076	0.542	−0.049	0.078	0.533
Thalassemia α	0.281	0.894	0.753	1.079	1.141	0.345
Thalassemia β	0.347	0.908	0.702	1.122	1.154	0.331
Gestational Thrombocytopenia	0.647	0.163	**< 0.001**	0.655	0.167	**< 0.001**

*^a^*For a specific disease group, a Bonferroni correction was applied to the significance level, α = 0.05 (two-sided), that divided 0.05 by the number of disease groups (10 groups). The results in bold are statistically significant (*p* < 0.05/10). The length of hospital stay and hospital costs were log-transformed before analysis. General linear models were used to estimate the coefficients of the pandemic group compared with the pre-pandemic group. The results in bold are statistically significant. Model 1 was not adjusted. Model 2 was adjusted for age, gestational age, gestational weight gain, Body mass index before pregnancy, nulliparity, assisted reproductive technology, and 10 disease groups (for “Total” only).

### Frequency of antibiotic prescribing before and during the COVID-19 pandemic

**FIGURE 3:**
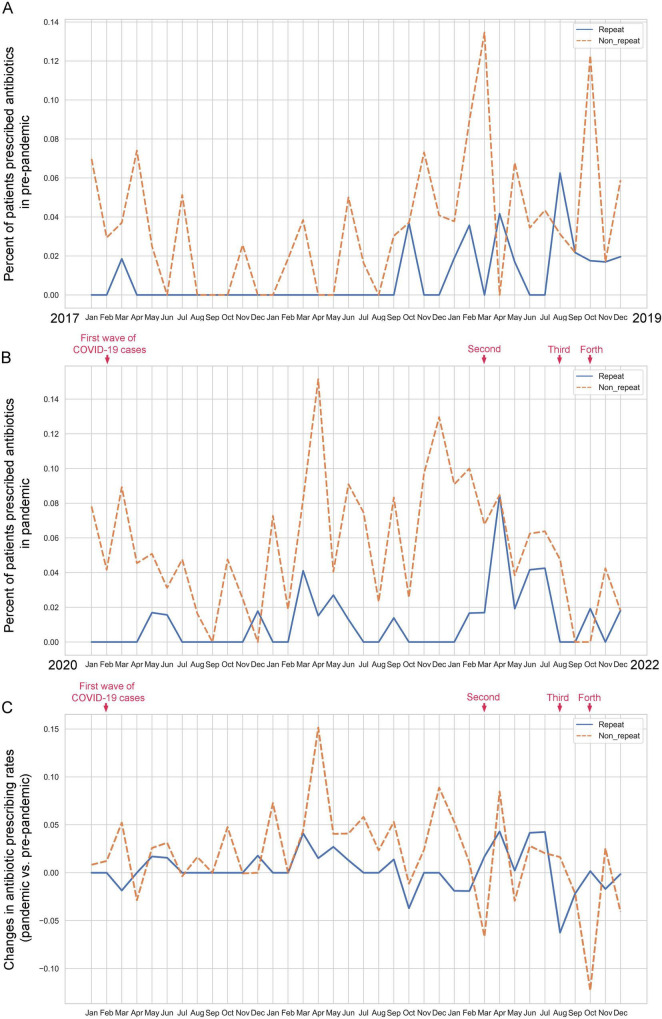
Comparison of monthly antibiotic prescribing patterns (repeat and non-repeat) for twin pregnancy inpatients in pre-pandemic and pandemic cohorts. **(A)** Percentage of monthly twin pregnancy inpatients prescribed antibiotics before the COVID-19 pandemic. **(B)** Percentage of monthly twin pregnancy inpatients prescribed antibiotics during the COVID-19 pandemic. **(C)** Change in the antibiotic prescribing rate of monthly twin pregnancy inpatients in pre-pandemic and pandemic cohorts.

The percentage of patients in the monthly pre-pandemic cohort receiving repeat prescriptions ranged from 0% to 6.3%, while those receiving non-repeat prescriptions ranged from 0% to 13.5% (Figure 3A). In the monthly pandemic cohort, the percentage of patients receiving repeat prescriptions ranged from 0% to 8.5%, and those receiving non-repeat prescriptions ranged from 0% to 15.2% (Figure 3B). Comparing the 3 years periods before and during the pandemic, the rate of twin pregnancy inpatients receiving antibiotic prescriptions increased for both repeat and non-repeat prescriptions during the COVID-19 pandemic (Figure 3C). The rise was particularly notable for non-repeat prescriptions, especially in the second year of the pandemic, reaching its peak in April 2021 (15.2%), possibly influenced by the significantly higher incidence of pregnancy complications during that month. After adjusting for seasonal influences (by subtracting pre-pandemic levels), we found that the rate of patients prescribed non-repeat antibiotics increased after each wave of the COVID-19 pandemic, except for the third wave (Figure 3C).

### Demographics of antibiotic prescribing in pre-pandemic and pandemic cohorts

**FIGURE 4:**
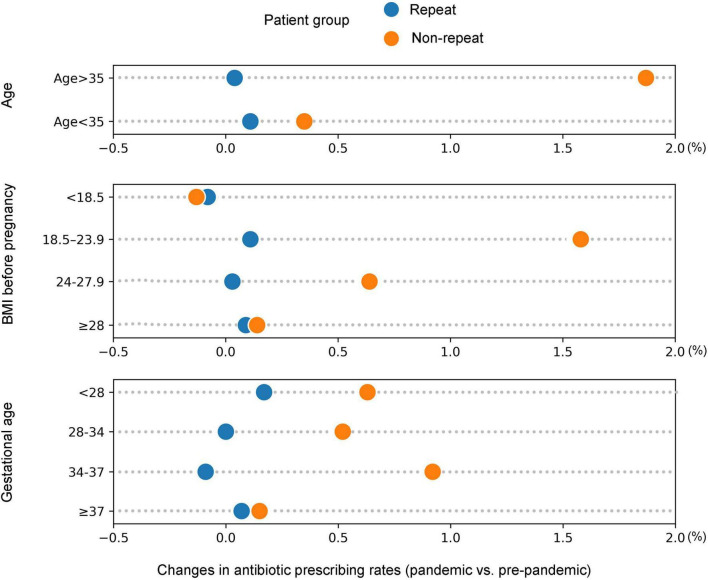
Changes in the rate of antibiotic prescribing (repeat and non-repeat) among patients in the pandemic versus pre-pandemic cohorts, categorized by the following demographic characteristics: age, BMI before pregnancy and gestational age.

The demographic characteristics of hospitalized twin pregnancies receiving repeat antibiotic prescriptions in pre-pandemic and pandemic cohorts were further analyzed (Table 3; see Supplementary Table 3 for non-repeat prescription demographics). Figure 4 visualizes the changes in repeat and non-repeat antibiotic prescribing rates in the pandemic versus pre-pandemic cohorts, categorized by patient demographics. Across most demographic categories, the change in repeat prescribing was either positive or similar to the overall pre-pandemic rate. The increase in non-repeat prescription rates during the pandemic was highly significant. Our analysis indicated a substantial rise in non-repeat antibiotic prescribing rates among patients over 35 years old, those with normal or higher body weight (BMI > 18.5), and those with preterm fetuses (< 37 weeks) during the COVID-19 pandemic. Specifically, the non-repeat antibiotic prescribing rate for patients over 35 years old increased by 1.87%.

**TABLE 3:** Characteristics of hospitalized twin pregnancies receiving repeat antibiotic prescriptions in pre-pandemic and pandemic cohorts.

Characteristics	Total (2017–2022)	Pre-pandemic (2017–2019)	Pandemic (2020–2022)
No. of cases	41	17	24
Ages, years	31.29 ± 4.03	32.69 ± 3.88	30.45 ± 3.96
Gestational age	31.36 ± 4.76	32.02 ± 3.87	31.13 ± 5.27
**Age groups (years)**			
< 35	32 (78.0%)	14 (82.4%)	18 (75.0%)
≥ 35	9 (22.0%)	3 (17.6%)	6 (25.0%)
Gestational weight gain, kg	–	15.92 ± 3.52	10.89 ± 5.45
**Body mass index before pregnancy**			
< 18.5	5 (12.2%)	3 (17.6%)	2 (8.3%)
18.5–23.9	29 (70.7%)	12 (70.6%)	17 (70.8%)
24–27.9	5 (12.2%)	2 (11.9%)	3 (12.5%)
≥ 28	2 (4.9%)	0 (0%)	2 (8.3%)
**Nulliparity**			
Yes	5 (12.2%)	3 (17.6%)	2 (8.3%)
No	36 (87.8%)	14 (82.4%)	22 (91.7%)
**Assisted reproductive technology**			
Using	9 (22.0%)	3 (17.6%)	2 (8.3%)
No using	32 (78.0%)	14 (82.4%)	22 (91.7%)
**Chorionicity**			
Dichorionic	33 (80.5%)	13 (76.5%)	20 (83.3%)
Monochorionic	8 (19.5%)	4 (23.5%)	4 (16.7%)
Length of hospital stay, days	12 (9,7.8)	14 (11, 21.2)	7 (6, 10.75)
Hospital costs, RMB^a^	11363.51 ± 3250.42	10714.71 ± 3574.81	11746.89 ± 3064.09

*^a^*The results were corrected by the consumer price index (CPI) in Chongqing, China (see Supporting Information: Supplementary Table 1 for details). Data are *n* (%) or mean (SD) or median (25th–75th percentile) unless otherwise specified.

### Patterns of antibiotic prescribing across patient clinical conditions in pre-pandemic and pandemic cohorts

**FIGURE 5:**
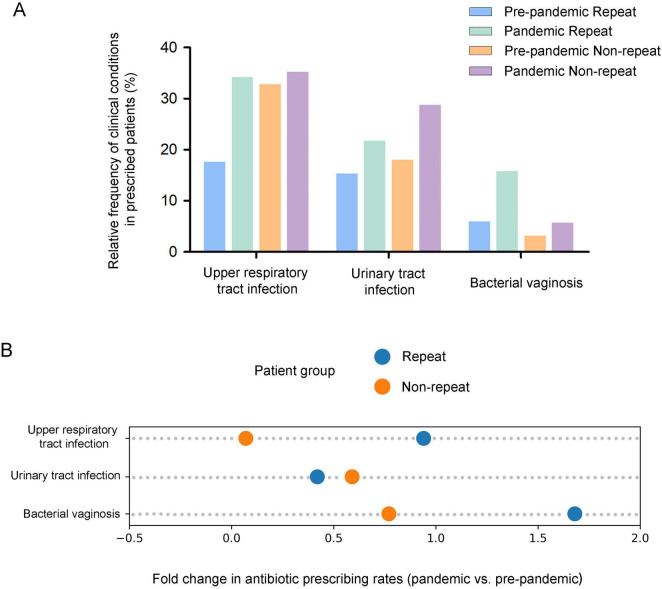
Comparison of antibiotic prescribing patterns (repeat and non-repeat) for the most common infections in twin pregnancy inpatients in pre-pandemic and pandemic cohorts. **(A)** Relative frequency of repeat and non-repeat antibiotic use in pre-pandemic and pandemic cohorts. **(B)** Fold change in the rate of repeat and non-repeat antibiotic use in pre-pandemic and pandemic cohorts.

Figure 5A shows the frequency of the three most common infections (upper respiratory tract infection, urinary tract infection, and bacterial vaginosis) among twin pregnancy patients prescribed repeat and non-repeat antibiotics in both pre-pandemic and pandemic groups. The highest antibiotic prescription rate was for upper respiratory tract infections, followed by urinary tract infections and bacterial vaginosis, in both the pre-pandemic and pandemic cohorts. Figure 5B displays the fold change in twin pregnancy inpatients with clinical conditions prescribed repeat and non-repeat antibiotics during the pandemic compared to the pre-pandemic period, reflecting changes in prescribing behavior and/or the incidence of these conditions. There was an increase in both repeat and non-repeat prescription rates for all infections during the pandemic. The most notable increases were a 1.68-fold rise in repeat prescriptions and a 0.77-fold rise in non-repeat prescriptions for twin pregnancy inpatients with bacterial vaginosis.

## Discussion

The impact of the first wave of the COVID-19 pandemic on pregnancies has been reported in numerous studies (4, 27, 28), but the long-term impact on these patients has not been investigated. Our study investigated the long-term impact of the COVID-19 pandemic on twin pregnancy hospitalizations, antibiotic prescribing, and maternal and neonatal health. We observed a marked increase in twin pregnancy hospitalizations during the pandemic, which gradually returned to pre-pandemic levels by April 2022. The pandemic group had a higher proportion of complications and ART use, as well as increased rates of pneumonia and NICU admissions for neonates. Furthermore, antibiotic prescribing for twin pregnancy inpatients also increased during the pandemic, with a more pronounced rise in non-repeat prescriptions, especially for older patients, those with higher BMI, and preterm fetuses. These findings provide a comprehensive understanding of the effects of the COVID-19 pandemic on twin pregnancy outcomes and antibiotic usage.

Previous studies reported disruptions in maternal health services (6–8) and a decline in inpatient admissions for non-COVID-19 patients during the COVID-19 pandemic (26, 29, 30). Contrary to these findings, this retrospective study observed a substantial increase in twin pregnancy hospitalizations after the pandemic began, with numbers gradually returning to pre-pandemic levels from April 2022. The most plausible explanation for this phenomenon is that medical resources were reallocated during the pandemic, leading maternal and child health hospitals to admit more patients with twin pregnancies and other pregnant women requiring special attention and treatment. Additionally, the Women and Children’s Hospital of Chongqing Medical University established a green channel for critically ill pregnant patients during the epidemic. Furthermore, increased maternal awareness of the risks associated with twin pregnancies during the pandemic may have led to earlier medical interventions. We found that mothers in the pandemic group were generally older than those in the pre-pandemic group (31.85 ± 3.87 years vs. 30.91 ± 3.90 years, pandemic vs. pre-pandemic). This trend may be due to women choosing to delay childbearing to focus on their careers and personal lives. The pandemic might have underscored the urgency of childbearing, especially for older women, prompting them to accelerate their reproductive plans. This urgency was reflected in the pronounced increase in twin pregnancies from March to August 2020, during the second year of the pandemic. Older women are more likely to use ART such as *in vitro* fertilization, which increases the likelihood of twin pregnancies (31, 32). Indeed, we observed a higher proportion of ART use during the 3 years of the COVID-19 pandemic (73.2% vs. 68.7%).

The reasons behind the increased proportion of pregnancy complications diagnosed in the pandemic group are multifaceted. First, quarantine policies, economic uncertainty, and health concerns during the pandemic heightened psychological stress and anxiety among pregnant women (4, 33). This heightened anxiety may have contributed to negative health behaviors, such as poor dietary choices and reduced adherence to recommended prenatal care, which can increase the risk of complications. Secondly, stay-at-home orders and restricted activities led to reduced physical activity, poorer dietary habits, and lifestyle changes, such as cessation of work, decreased exercise, and unhealthy eating patterns, which could partly explain the observed increase in pregnancy complications, including gestational diabetes and hypertension (4, 34). Finally, the increased use of ART during the pandemic, which is associated with a higher risk of pregnancy complications, also contributed to this trend (35). Consistent with previous research (36), there was an increase in the rate of pneumonia (5.7% vs. 3.8%) and NICU admissions (43.7% vs. 13.9%) for neonates during the pandemic period. This may also be related to the increased maternal age, the higher use of ART, delays in prenatal car, and lifestyle changes. Therefore, it is essential to provide mental health support, encourage safe physical activity, promote nutritional guidance, enhance ART monitoring, expand telehealth services for continuous prenatal care and early intervention, and address both psychological and lifestyle factors alongside medical interventions to reduce pregnancy complications during times of crisis.

Antibiotic resistance poses a formidable challenge to public health, making the surveillance of antibiotic usage crucial for developing effective stewardship policies (37, 38). Comparative studies can reveal whether changes in infection control strategies during the pandemic have altered antibiotic usage patterns. Understanding these changes can help hospitals and public health institutions identify and address potential infection control issues, optimizing future responses. To investigate the impact of COVID-19 pandemic-related pressures, we analyzed the prescribing patterns (repeat/non-repeat) among monthly cohorts of twin pregnancy inpatients before and during the COVID-19 pandemic. Comparing data from the 3 years before and during the pandemic helps exclude the potential influence of seasonal antibiotic use. It has been documented that there were instances of improper antibiotic use in both healthcare institutions and communities during the pandemic (39). However, some studies have reported a decline in antibiotic prescribing during this period (23, 24). Our results showed that for both repeat and non-repeat prescribing, the rate of twin pregnancy inpatients receiving antibiotic prescriptions increased during the 3 years period of the COVID-19 pandemic. Notably, the proportion of non-repeat prescribing showed a more pronounced increase compared to repeat prescribing, especially in the second year of the pandemic. This suggests that non-repeat antibiotic prescribing rates were more significantly affected by COVID-19-related changes among these patients.

A more thorough analysis of the demographic characteristics of hospitalized twin pregnancies cohorts could provide more insight and better inform interpretations. Specifically, our analysis of prescriptions indicated a significant increase in the rate of non-repeat antibiotic prescribing among patients aged over 35 years, those with normal or higher body weight (BMI > 18.5), and those carrying preterm fetuses (< 37 weeks) during the COVID-19 pandemic. This is likely due to the fact that premature twin pregnancies among older and heavier women tend to have lower immunity, rendering them more prone to complications and often presenting with more complex clinical conditions, which consequently increase the risk of infections (40, 41). We examined the changes in antibiotic prescribing before and during COVID-19 for the three most common infections in twin pregnancies: upper respiratory tract infections, urinary tract infections, and bacterial vaginosis. Our results showed an increase in repeat/non-repeat antibiotic prescribing for all three infections during the COVID-19 pandemic. The SARS-CoV-2 virus itself can cause respiratory infections, and pregnant women, especially those with twin pregnancies, were at higher risk if infected (42, 43). To prevent secondary bacterial infections triggered by COVID-19, doctors might prescribe various antibiotics to cover a broader range of pathogens, ensuring more comprehensive protection. Compared to the pre-pandemic cohorts, the rate of repeated antibiotic prescriptions for bacterial vaginosis showed a remarkable surge during the COVID-19 pandemic, increasing by 1.68-fold. In cases of bacterial vaginosis, the presence of resistant bacterial strains may necessitate the use of multiple courses of antibiotics to manage and overcome resistant infections, ensuring effective treatment. In general, non-repeat prescribing showed a more significant rise during the 3 years period of the COVID-19 pandemic, suggesting that clinician caution regarding secondary infections, particularly respiratory tract infections and bacterial vaginosis, was a key driver. Furthermore, the increased use of ART during the pandemic, which is associated with a higher risk of infections, may have also contributed to the increase in antibiotic prescribing. Understanding the reasons behind these prescribing trends is critical for future infection control strategies and antibiotic stewardship.

It is believed that this is the first study investigating the long-term impact of the COVID-19 pandemic on twin pregnancy inpatients populations. Our study provides in-depth insights into the pandemic’s effects on health outcomes and antibiotic use among twin pregnancy inpatients, aiming to optimize clinical treatment, enhance healthcare resource management, and promote maternal and child health and public health safety. However, limitations still exist in this study. First of all, as a retrospective analysis, there is potential for selection bias. Additionally, the study was conducted at a single center with a relatively small sample size, limiting the generalizability of our findings. Furthermore, due to insufficient data, we were unable to evaluate the impact of COVID-19 infection status, vaccination coverage, and lockdown measures on twin pregnancy inpatients. Therefore, multicenter prospective studies are needed to validate our observations and investigate the impact of COVID-19 vaccines or COVID-19 infections on patients with twin pregnancies.

## Conclusion

This study provides an in-depth exploration of the long-term impact of the COVID-19 pandemic on twin pregnancy inpatients, including hospitalization trends, maternal and neonatal health, and antibiotic prescribing patterns. The results show a significant increase in twin pregnancy hospitalizations during the pandemic. Patients in the pandemic cohort were generally older and exhibited a higher prevalence of pregnancy complications, along with an increased rate of ART use. Neonatal outcomes also worsened, with higher rates of pneumonia and NICU admissions. Additionally, there was an increase in antibiotic prescriptions during the pandemic, particularly non-repeat prescriptions, which were more prominent among older patients, those with higher BMI, and those carrying preterm fetuses. This study offers a comprehensive understanding of the effects of the COVID-19 pandemic on twin pregnancies, with findings that may be particularly relevant to high-volume maternity hospitals. However, larger, multicenter studies are needed to confirm these trends. Our research also provides valuable insights and guidance for responding to future public health crises. To optimize antimicrobial stewardship and strengthen maternal healthcare systems during future pandemics, it is essential for obstetricians and policymakers to implement strategies such as telemedicine, standardized antibiotic prescribing protocols, enhanced monitoring, as well as providing mental health support, promoting safe physical activity, and offering nutritional guidance.

## Data Availability

The original contributions presented in this study are included in this article/Supplementary material, further inquiries can be directed to the corresponding authors.

## Footnotes

1
https://wsjkw.cq.gov.cn/
