# Supplementary material for: Three-year impact of COVID-19 pandemic on hospitalized twin pregnancies: evaluation of characteristics and changes in antibiotic prescribing
Source: Front Med (Lausanne). 2025 Jun 18;12:1546013. doi: 10.3389/fmed.2025.1546013 (PMC12213898; doi:10.3389/fmed.2025.1546013)
Supplement: Supplementary file 1 [file Table_1.DOCX]

Supplementary Material

# Supplementary Tables

**Table S1. Consumer price index (CPI) of medical care in Chongqing, China (2017-2022).**

| **Year** | **CPI (compared with last year)** |
| --- | --- |
| 2017 | Reference |
| 2018 | 0.1% |
| 2019 | 1.7% |
| 2020 | 1.3% |
| 2021 | -1.0% |
| 2022 | 1.1% |

Data source: Chongqing Bureau of Statistics

<http://tjj.cq.gov.cn/zwgk_233/fdzdgknr/tjxx/>

**Table S2 Comparison of length of hospital stays and hospital costs for fetal diagnosis.**

|  | **Length of hospital stays** | | | **Hospital costs** | | |
| --- | --- | --- | --- | --- | --- | --- |
|  | **β** | **SE** | ***P ^a^*** | **β** | **SE** | ***P ^a^*** |
| **Model 1** |  |  |  |  |  |  |
| **Total** | 0.493 | 0.269 | 0.067 | -0.338 | 0.331 | 0.307 |
| 1-Minutes Apagar < 7 | 0.125 | 0.335 | 0.710 | 0.219 | 0.341 | 0.522 |
| Admission to pediatrics | -0.287 | 0.091 | **<0.001** | -2.123 | 0.095 | **<0.001** |
| Admission to NICU | 4.520 | 0.175 | **<0.001** | 4.528 | 0.181 | **<0.001** |
| Hypoglycemia | -1.782 | 0.278 | **<0.001** | -1.668 | 0.290 | **<0.001** |
| Hyperbilirubinemia | -1.293 | 0.143 | **<0.001** | -1.307 | 0.147 | **<0.001** |
| Respiratory distress | -1.573 | 0.248 | 0.607 | -1.159 | 0.255 | 0.180 |
| Respiratory failure | -0.121 | 0.235 | **<0.001** | -0.327 | 0.244 | **<0.001** |
| Pneumonia | 3.283 | 0.227 | **<0.001** | 3.276 | 0.237 | **<0.001** |
| Lower gastrointestinal bleeding | -0.065 | 0.260 | 0.796 | -0.207 | 0.275 | 0.452 |
| Hypoproteinemia | -0.359 | 0.389 | 0.803 | -0.261 | 0.415 | 0.530 |
| Hyperelastic acidemia | 0.079 | 0.289 | 0.357 | 0.039 | 0.307 | 0.900 |
| Necrotizing enterocolitis | -0.102 | 0.396 | 0.784 | 0.053 | 0.410 | 0.896 |
| **Model 2** |  |  |  |  |  |  |
| **Total** | 0.119 | 0.280 | 0.671 | -0.430 | 0.389 | 0.204 |
| 1-Minutes Apagar < 7 | 0.286 | 0.267 | 0.283 | 0.333 | 0.271 | 0.219 |
| Admission to pediatrics | -0.809 | 0.072 | **<0.001** | -0.841 | 0.075 | **<0.001** |
| Admission to NICU | 1.731 | 0.091 | **<0.001** | 1.694 | 0.092 | **<0.001** |
| Hypoglycemia | -0.315 | 0.201 | 0.117 | -0.196 | 0.209 | 0.347 |
| Hyperbilirubinemia | -0.080 | 0.102 | 0.431 | 0.094 | 0.104 | 0.368 |
| Respiratory distress | -0.478 | 0.155 | **0.002** | -0.527 | 0.160 | **0.001** |
| Respiratory failure | -0.198 | 0.146 | 0.176 | -0.306 | 0.151 | 0.042 |
| Pneumonia | 1.715 | 0.177 | **<0.001** | 1.756 | 0.186 | **<0.001** |
| Lower gastrointestinal bleeding | 0.079 | 0.205 | 0.699 | -0.070 | 0.215 | 0.744 |
| Hypoproteinemia | -0.219 | 0.286 | 0.445 | -0.073 | 0.309 | 0.814 |
| Hyperelastic acidemia | -0.162 | 0.227 | 0.475 | -0.136 | 0.238 | 0.567 |
| Necrotizing enterocolitis | 0.058 | 0.311 | 0.852 | 0.2007 | 0.326 | 0.526 |

***Note:*** The length of hospital stay and hospital costs were log‐transformed before analysis. General linear models were used to estimate the coefficients of the pandemic group compared with the pre-pandemic group. The results in bold are statistically significant.

Model 1 was not adjusted.

Model 2 was adjusted for age, gestational age, gestational weight gain, Body mass index before pregnancy, nulliparity, assisted reproductive technology, and 12 disease groups (for “Total” only).

**a:** For a specific disease group, a Bonferroni correction was applied to the significance level, α = 0.05 (two‐sided), that divided 0.05 by the number of disease groups (12 groups). The results in bold are statistically significant (p < 0.05/12).

**Table S3 Characteristics of hospitalized twin pregnancies receiving un-repeat antibiotic prescriptions in pre-pandemic and pandemic cohorts.**

| **Characteristics** | **Total (2017–2022)** | **Pre-pandemic (2017–2019)** | **Pandemic (2020–2022)** |
| --- | --- | --- | --- |
| **No. of cases** | 183 | 61 | 122 |
| **Ages, years** | 30.55±3.91 | 30.46±4.01 | 30.61±3.87 |
| **Gestational age** | 33.79±3.92 | 34.21±3.12 | 33.58±4.27 |
| **Age groups (years)** |  |  |  |
| < 35 | 160 (87.4%) | 54 (88.5%) | 106 (86.9%) |
| ≥ 35 | 23 (12.6%) | 7 (11.5%) | 16 (13.1%) |
| **Gestational weight gain, kg** | 15.10±5.51 | 16.61±5.06 | 14.13±5.60 |
| **Body mass index before pregnancy** |  |  |  |
| < 18.5 | 15 (8.2%) | 8 (13.1%) | 7 (5.7%) |
| 18.5 – 23.9 | 127 (69.4%) | 42 (69.7%) | 85 (69.7%) |
| 24 - 27.9 | 38 (20.8%) | 11 (18.0%) | 27 (22.1%) |
| ≥ 28 | 3 (1.6%) | 0 (0%) | 3 (2.5%) |
| **Nulliparity** |  |  |  |
| Yes | 28 (15.3%) | 6 (9.8%) | 22 (18.0%) |
| No | 155 (84.7%) | 55 (90.2%) | 100 (82.0%) |
| **Assisted reproductive technology** |  |  |  |
| Using | 43 (23.5%) | 16 (26.2%) | 27 (22.1%) |
| No using | 140 (76.5%) | 45 (73.8%) | 95 (77.9%) |
| **Chorionicity** |  |  |  |
| Dichorionic | 155 (84.7%) | 53 (86.9%) | 102 (83.6%) |
| Monochorionic | 28 (15.3%) | 8 (13.1%) | 20 (16.4%) |
| **Length of hospital stay, days** | 6 (4, 9) | 5.5 (4, 7) | 6 (4, 11) |
| **Hospital costs, RMB ^a^** | 11611.45±4170.37 | 11899.51±3980.59 | 11460.82±4276.49 |

Note: Data are n (%) or mean (SD) or median (25th–75th percentile) unless otherwise specified.

a: The results were corrected by the consumer price index (CPI) in Chongqing, China (see Supporting Information: Table S1 for details).
